# Prevalence and impact of sleep-related breathing disorder in multiple system atrophy patients: a cross-sectional study and meta-analysis

**DOI:** 10.3389/fneur.2024.1440932

**Published:** 2024-08-20

**Authors:** Hui Wang, Ting Zhang, Wenhui Fan, Yanming Xu

**Affiliations:** ^1^Department of Neurology, Sichuan Taikang Hospital, Chengdu, Sichuan, China; ^2^Department of Neurology, West China Hospital of Sichuan University, Chengdu, Sichuan, China

**Keywords:** multiple system atrophy, sleep related breathing disorder, prevalence, motor symptoms, nonmotor symptoms

## Abstract

**Objective:**

Sleep-related breathing disorder (SRBD) is a prevalent non-motor symptom in multiple system atrophy (MSA). However, the reported prevalence of SRBD in MSA from different studies has shown inconsistency. Additionally, only one study has examined the impact of SRBD on both motor and non-motor symptoms in MSA.

**Methods:**

Cross-sectional study of 66 patients with probable MSA from China. SRBD was ascertained with polysomnography (PSG). All the MSA individuals were assessed using the Epworth Sleepiness Scale (ESS), Unified Multiple-System Atrophy Rating Scale (UMSARS), Hamilton Depression Scale (HAMD), Hamilton Anxiety Scale (HAMA), the Mini-mental State Examination (MMSE), Non-Motor Symptoms Scale (NMSS), and Pittsburgh Sleep Quality Index (PSQI). Moreover, a meta-analysis was conducted by searching studies related to MSA and SRBD in PubMed, Web of Science, Embase, and Cochrane databases. Data were pooled as necessary to calculate prevalence of SBRD with 95% confidence intervals (CI).

**Results:**

Our study included 66 patients with MSA, 52 of whom had a diagnosis of SRBD (78.8%). There were no significant differences between the MSA with SRBD and without SRBD groups on the age, sex, disease onset, disease duration, UMSARS I, II, and IV, the NMSS, the HAMA, HAMD, the ESS the FSS, the MMSE, and the PSQI scales. However, MSA patients with SRBD having a significant higher obstructive apnea index and percentage of snoring during sleep than MSA patients without SRBD [10.0 (4.1–10.6) vs. 0.1 (0–0.3), and 8.3 (5.1–12.2) vs. 4.2 (0–7.5)]. Also, between the two groups, the mean and minimum oxygen concentrations during sleep were lower in MSA patients with SRBD than in those without SRBD [93.7 (93–95) vs. 95.5 (95.8–97), *p* = 0.001] and [83.9 (81.2–89.0) vs. 90.3 (89.8–93.3), *p* = 0.000]. The primary search strategy identified 701 articles, with 10 meeting the inclusion criteria. The overall prevalence of SRBD in a combined sample of 295 MSA patients was found to be 60.5% (95% CI, 43.2–76.5%). Further analysis revealed that the prevalence of SRBD in MSA patients in Asia was 79.2% (95% CI, 54.7–96.3%), which was higher than that in Europe (41.6, 95% CI, 32–51.5%).

**Conclusion:**

The study found a prevalence of 78.8% of SRBD in MSA patients, with a notably higher prevalence in Asia compared to Europe. The majority of SRBD cases in MSA were attributed to obstructive apnea. Furthermore, the presence of SRBD did not show a significant impact on the motor and non-motor symptoms of MSA patients.

## Introduction

Multiple system atrophy (MSA) was first proposed by Graham and Oppenheimr in 1969 as an alpha-synucleinopathy disease characterized by poor response to levodopa, autonomic dysfunction and/or cerebellar ataxia. It can be classified into two types: MSA-P (predominant Parkinsonism) and MSA-C (predominant cerebellar ataxia), based on the main motor symptoms (1). MSA-P type presents Parkinson’s syndrome as the main symptom, while MSA-C type shows cerebellar ataxia disorder as the main symptom. Further research on MSA has revealed that it not only exhibits typical motor symptoms but also numerous non-motor symptoms, such as sleep-related breathing disorders (1).

Sleep-related breathing disorder (SRBD) is a common sleep disorder in patients with neurodegenerative diseases, including Alzheimer’s disease (AD), Parkinson’s disease (PD), dementia with Lewy bodies, MSA, hereditary ataxia, and amyotrophic lateral sclerosis (ALS) (2). SRBD typically manifests as obstructive sleep apnea (OSA), central sleep apnea (CSA), irregular breathing, apnea, Cheyne-Stokes breathing pattern, and stridor (2). Certain episodes of sleep breathing disorders can impact patient safety, with issues like nocturnal wheezing potentially leading to sudden nocturnal death (3). Wheezing in the early stages of MSA has been identified as an independent risk factor for shorter survival in MSA patients (4). Moreover, central and peripheral respiratory disturbances are common in MSA patients, occurring during both sleep and wakefulness. These disturbances can include intermittent involuntary gasping, periodic or irregular breathing, abnormal hypoxic and hyperventilatory responses, respiratory failure, and stridor (2).

Sleep-related breathing disorder are a common non-motor symptom in patients with MSA. However, current reports on the prevalence of SRBD in MSA show varying results, with previous studies indicating occurrence rates ranging from approximately 24.9–100% among MSA patients (5–14). Research on the impact of SRBD on both motor and non-motor symptoms in MSA is limited. Only one study published in 2017 examining the effects of SRBD on MSA patients revealed that those with SRBD exhibited more severe motor deficits, depressive symptoms, and frontal lobe dysfunction compared to those without SRBD. Additionally, they experienced more daytime sleepiness, sleep deprivation, longer average sleep duration, and OSA (9).

This study aims to further investigate the prevalence of SRBD in MSA and its effects on motor and non-motor symptoms. Furthermore, a meta-analysis of previously published studies on SRBD prevalence in MSA patients will be conducted to discuss the factors contributing to the wide range of reported SRBD prevalence in MSA.

## Methods

This cross-sectional study included 66 consecutive patients with probable MSA who were admitted to the Department of Neurology at West China Hospital and Sichuan Taikang Hospital between 2016 and 2024. Diagnosis was made by neurologists based on second consensus statement on the diagnosis of MSA proposed by Gilman et al. (15). Apnea was defined as a ≥90% decrease in the airflow signal from baseline for ≥10 s, while hypopneas were diagnosed as a ≥ 30% decrease in airflow lasting ≥10 s, associated with either ≥3% desaturation from the prevent baseline or an arousal. Irregular breathing was defined as irregular respiratory rhythm, which is characterized by alternating depths or uneven speeds (16). Blood oxygen was defined as the oxygen saturation measured by finger oximetry. Periodic limb movements were defined in the 2007 AASM Manual for the Scoring of Sleep and Associated Events (17). SRBD were identified by an Apnea-Hypopnea Index (AHI) > 5/h, with severity categorized as mild (AHI: 5–15/h), moderate (AHI: 15–30/h), and severe (AHI > 30/h) (17). Polysomnography (PSG) was conducted at the Sleep Medicine Center of West China Hospital.

The study was approved by the Ethics Committees of West China Hospital, Sichuan University (2020-842), and all patients provided written consent. Patients meeting the diagnostic criteria for probable MSA, completing relevant questionnaires and PSG, and showing no brain injury on MRI were included. Exclusion criteria were the use of medications affecting sleep, cognitive impairment, acute psychosis, or critical illness.

Demographic and clinical characteristics were recorded for all MSA patients, with “disease onset” defined as the initial motor or autonomic symptom presentation and “disease duration” as the time from symptom onset to study enrollment. Various questionnaires were used to evaluate clinical characteristics in the patients. The Non-Motor Symptoms Scale (NMSS, nine domains) was used to evaluate the severity of non-motor symptoms. The severity of depression and anxiety were assessed, respectively, using the Hamilton Depression Rating Scale (HAMD, 17 items) and Hamilton Anxiety Rating Scale (HAMA). Patients were also evaluated using the Fatigue Severity Scale (FSS) and Epworth Sleepiness Scale (ESS). Severity of motor symptoms was assessed using the Unified Multiple System Atrophy Rating Scale (UMSARS); cognitive function, using the Mini-mental State Examination (MMSE); non-motor symptoms, Non-Motor Symptoms Scale (NMSS); and sleep quality, using the Pittsburgh Sleep Quality Index (PSQI).

### Overnight video-PSG

Overnight video-PSG began for each patient at 22:00 every night in a quiet room with appropriate lighting and temperature, and it consisted of continuous recordings by electroencephalography (F4–M1, C4–M1, O2–M1, F3–M2, C3–M2, and O1–M2), electro-oculography (ROC–M1, LOC–M2), submental electromyography, right and left anterior tibialis surface electromyography, and electrocardiography. Overnight PSG was performed with video and audio in the Sleep Medicine Center of West China Hospital at Sichuan University. PSG was used to monitor the following indices: video, audio, electroencephalography, blood oxygen, oral-nasal airflow, thoracic and abdominal breathing, electrocardiography, eye movement, and mandible and limb electromyography. PSG results were scored by sleep technicians and interpreted by sleep specialists. Sleep staging, respiratory events, arousal, and limb movements were evaluated according to the guidelines for scoring sleep and related events from the American Academy of Sleep Medicine (18).

The following sleep variables were analyzed: sleep latency (SL); REM sleep latency; sleep efficiency (SE); total sleep time; percentage of total time spent in stage N1, N2, and N3 or REM sleep; wake after sleep onset (WASO); apnea–hypopnea index (AHI); Obstructive apnea index; Central apnea index; average/minimum oxygen saturation (SaO_2_); and periodic leg movement index (PLMI).

### Statistical analysis

Data were analyzed using SPSS 19.0 (IBM, Chicago, IL, United States). Continuous data showing a normal distribution were expressed as mean ± standard deviation (SD), while continuous skewed data were reported as median (interquartile range). Inter-group differences were assessed for significance using Student’s *t* test. Inter-group differences in continuous skewed data were assessed using a Mann–Whitney U test. Differences in categorical data were assessed using the chi-squared test. Logistic regression analysis was used to estimate the odds ratio (OR) and 95% CI after adjusting for potential confounding factors, including quantitative variables (age and disease duration) and categorical variables (sex, smoking, alcohol consumption, and MSA sub-type). Differences associated with *p* < 0.05 were statistically significant.

### Meta-analysis methods

#### Searching strategy

This meta-analysis was conducted following the guidelines of the Preferred Reporting Items for Systematic Reviews and Meta-Analyses (PRISMA) (19). The Cochrane Collaboration definition for systematic review and meta-analysis was strictly followed. Two authors (HW and TZ) independently searched Medline via PubMed, Web of Science, Embase via embase.com, and Cochrane databases for original published studies on the clinical manifestations of MSA patients with or without SRBD. Inclusion criteria were studies published in English before April 2, 2024.The search string was as follows: “Sleep Apnea Syndromes” OR “Apnea Syndrome, Sleep” OR “Apnea Syndromes, Sleep” OR “Sleep Apnea Syndrome” OR “Sleep Hypopnea” OR “Hypopnea, Sleep” OR “Hypopneas, Sleep” OR “Sleep Hypopneas” OR “Apnea, Sleep” OR “Apneas, Sleep” OR “Sleep Apnea” OR “Sleep Apneas” OR “Sleep Apnea, Mixed Central AND Obstructive” OR “Mixed Central AND Obstructive Sleep Apnea” OR “Sleep Apnea, Mixed” OR “Mixed Sleep Apnea” OR “Mixed Sleep Apneas” OR “Sleep Apneas, Mixed” OR “Hypersomnia with Periodic Respiration” OR “Sleep-Disordered Breathing” OR “Breathing, Sleep-Disordered” OR “Sleep Disordered Breathing” OR “sleep-related breathing disorders” OR “sleep related breathing disorders” OR ““Sleep Apnea Syndromes” OR “Sleep Apnea, Obstructive” OR “Apneas, Obstructive Sleep” OR “Obstructive Sleep Apneas” OR “Sleep Apneas, Obstructive” OR “Obstructive Sleep Apnea Syndrome” OR “Obstructive Sleep Apnea” OR “OSAHS” OR “Syndrome, Sleep Apnea, Obstructive” OR “Sleep Apnea Syndrome, Obstructive” OR “Apnea, Obstructive Sleep” OR “Sleep Apnea Hypopnea Syndrome” OR “Syndrome, Obstructive Sleep Apnea” OR “Upper Airway Resistance Sleep Apnea Syndrome” OR “Syndrome, Upper Airway Resistance, Sleep Apnea” AND “Atrophy, Multiple System” OR “Multiple System Atrophies” OR “Multisystemic Atrophy” OR “Atrophies, Multisystemic” OR “Atrophy, Multisystemic” OR “Multisystemic Atrophies” OR “Multiple System Atrophy Syndrome” OR “Multisystem Atrophy” OR “Atrophies, Multisystem” OR “Atrophy, Multisystem” OR “Multisystem Atrophies.”

#### Study selection criteria

Articles were initially screened based on their titles and abstracts, with full text consulted when necessary. Patients were diagnosed with SRBD according to objective instruments, including polysomnography (PSG), Embletta and Apnea Link sleep monitoring devices. In addition, SRBD was diagnosed in the occurrence of an AHI > 5/h (17). Inclusion criteria were (1): original data on SRBD and clinical symptoms of MSA (2), patients diagnosed with probable MSA, (3) and sufficient data to calculate the prevalence of SRBD in MSA patients and the impact of SRBD on MSA patients.

Exclusion criteria were (1): reviews, editorials, conference abstracts, letter or case reports (2); focus solely on SRBD characteristics, pathogenic mechanisms, or MSA management with SRBD (3); comparisons between MSA and other synucleinopathies (4); insufficient data for meta-analysis (5); non-English articles (6); or studies not involving human subjects. Discrepancies in article inclusion were resolved by a third author (WF).

#### Data extraction and study quality assessment

The data extracted from the original articles included the surname of the first author, publication year, country, sample size, prevalence of SRBD, mean age of patients, sex, disease onset, disease duration. For longitudinal studies, only baseline data were extracted.

The quality of the included studies was evaluated using the Newcastle-Ottawa Scale (NOS) for case–control and cohort studies, as well as the Agency for Healthcare Research and Quality guideline (AHRQ) for cross-sectional studies (20, 21). Any discrepancies were resolved through consensus among all authors.

#### Statistical analysis

The STATA software version 16.0 was used for statistical analysis. The primary outcome measure was frequency of SRBD in MSA as reported in prevalence (%). The pooled prevalence of SRBD and 95% confidence intervals were obtained by using a DerSimonian-Laird random-effects model with double arcsine transformation (22). A *p* value equal to or less than 0.05 was considered statistically significant. The heterogeneity across studies was evaluated using Cochrane’s *I*^2^ values. *I*^2^ > 75% was defined as high heterogeneity, 50% < *I*^2^ < 75% as moderate heterogeneity, 25% < *I*^2^ < 50% as low heterogeneity, and *I*^2^ < 25% as homogeneity. The Begg’s and Egger’s test was created to detect publication biases.

## Results

Polysomnography was completed in 66 MSA patients, and these 66 patients were included in this study, of which 52 (78.8%) reached the diagnosis of SRBD (AHI >5/h), including 31 males and 21 females.

Among the 52 patients with SRBD, 19 patients had mild (AHI: 5–15/h), 15 patients had moderate (AHI: 15–30/h), and 18 patients had severe (AHI: >30/h), and the three types accounted for 36.5, 28.8, and 34.6% of all patients, respectively.

As shown in Table 1, in this study, 66 patients diagnosed with MSA were categorized into two groups based on the presence of SRBD. The analysis focused on comparing motor and non-motor symptoms between these two groups. The study found no statistically significant variances between the groups in terms of motor symptom severity assessed by the UMSARS scale, non-motor symptoms evaluated through the NMSS scale, anxiety and depression levels measured by the HAMA and HAMD scales, somnolence assessed by the ESS scale, fatigue evaluated using the FSS scale, cognitive functioning measured by the MMSE scale, as well as assessments of nocturnal sleep functioning PSQI scales (*p* > 0.05). Notably, 71.2% of MSA patients exhibited periodic leg movements, with 35 of them also displaying SRBD.

**Table 1:** Comparison of motor and non-motor symptoms in MSA patients with and without SRBD.

Characteristics	All MSA patients (*n* = 66)	With SRBD (*n* = 52)	Without SRBD (*n* = 14)	*p*
Male/Female	40/26	31/21	9/5	0.753
Age(years)	63.05 ± 10.13	63.69 ± 9.60	60.64 ± 11.58	0.325
Disease onset (year)	60.15 ± 10.3	60.74 ± 9.91	57.93 ± 11.01	0.370
Disease duration (year)	2.79 ± 2.78	2.61 ± 1.86	2.84 ± 2.98	0.962
UMSARS I	13.23 ± 5.8	13.06 ± 5.74	13.86 ± 5.99	0.648
UMSARS II	16.52 (12–20.25)	16.33 ± 6.12	17.29 ± 6.23	0.638
UMSARS IV	1.74 ± 0.99	1.65 ± 0.84	2.00 ± 1.36	0.751
NMSS	49.35 ± 26.4	49.79 ± 26.23	47.71 ± 28.16	0.797
HAMA	11.14 ± 7.88	10.84 ± 7.29	12.21 ± 9.64	0.804
HAMD	10.28 ± 6.99	9.78 ± 6.14	12.07 ± 9.23	0.642
ESS	7.11 (2.75–11.00)	7.23 (2.25–11.75)	6.64 (2.75–9.00)	0.571
FSS	21.71 (11–30)	22.98 (11–34)	17 (12.50–21.25)	0.723
MMSE	24.58 (22–29)	24.13 (21.25–28)	26.21 (24.50–29.00)	0.172
PLMS (%)	47 (71.2)	35 (67.3)	12 (85.7)	0.318
PSQI	6.64 (3.0–9.5)	6.90 (3–9)	5.64 (1.75–10.00)	0.194

ESS, Epworth Sleepiness Scales; FSS, Fatigue severity scale; HAMA, Hamilton anxiety scale; HAMD, Hamilton depression scale; MSA, Multiple system atrophy; MMSE, Mini-mental state examination; NMSS, Non-motor symptoms scale; PLMS, Periodic limb movements syndrome; PSQI, Pittsburgh sleep quality index; SRBD, Sleep related breathing disordered; and UMSARS, Unified multiple-system atrophy rating scale.

Values are *n* (%), mean ± SD or median (interquartile range).

As illustrated in Table 2, all 66 patients diagnosed with MSA in this particular study underwent PSG. There were no significant variations observed between MSA patients with SRBD and those without SRBD in terms of various sleep parameters such as total sleep time (TST), sleep efficiency (SE), sleep latency (SL), sleep structure (N1, N2, and N3, and the percentage of REM phase in the entire sleep cycle), arousal index, wake after sleep onset (WASO), central apnea index, and periodic leg movement index (PLMI). However, it was noted that MSA patients with SRBD exhibited a notably higher obstructive apnea and hypopnea index compared to MSA patients without SRBD [10.0 (4.1–10.6) vs. 0.1 (0–0.3) and 14.7(8.5–19.3) vs. 2.3(1.3–2.9), *p* = 0.000]. Furthermore, MSA patients with SRBD displayed a significantly higher occurrence of snoring during sleep in comparison to those without SRBD [8.3 (5.1–12.2) vs. 4.2(0–7.5), *p* = 0.015]. Additionally, compared with MSA patients without SRBD, MSA patients with SRBD had lower mean and minimum oxygen concentrations and higher oxygen desaturation index during sleep {[93.7 (93–95) vs. 95.5 (95.8–97), *p* = 0.001], [83.9 (81.2–89.0) vs. 90.3 (89.8–93.3), *p* = 0.000], and 19.9 (10.9–36.6) vs. 2.5(1.5–4.0)}.

**Table 2:** Comparison of PSG measurements in MSA patients with and without SRBD.

Variables	All MSA patients (*n* = 66)	With SRBD (*n* = 52)	Without SRBD (*n* = 14)	*p*
TST	337.0 ± 90.2	334.3 ± 86.4	346.9 ± 106.0	0.647
SE, %	66.0 ± 17.3	65.9 ± 16.9	66.4 ± 19.6	0.920
SL, min	22.9(5–28)	22.8(5.5–26.1)	23.1(2.4–33.9)	0.660
N1, %	32.2(30.6–42.35)	33.8(19.6–43.6)	25.9(14.5–34.6)	0.134
N2, %	48.1 ± 14.6	47.88 ± 15.3	48.89 ± 12.0	0.819
N3, %	1.5(0–0.32)	1.1(0–0.3)	3.0(0–2.7)	0.369
REM sleep, %	18.3 ± 3.8	17.19 ± 6.9	22.21 ± 9.8	0.890
WASO, min	151.1 ± 83.9	150.8 ± 81.2	152.1 ± 96.4	0.962
Arousal index, /h	19.7(13.6–23.6)	20.6(11.5–26.0)	16.3(10.5–21.5)	0.246
OA index, /h	7.6(0–9)	10.0(4.1–10.6)	0.1(0–0.3)	**0.000**
CA index, /h	0.4(0–0.4)	0.5(0–0.5)	0.1(0–0.1)	0.122
hypopnea index, /h	9.6 (3.4–17.3)	14.7(8.5–19.3)	2.3(1.3–2.9)	**0.000**
Total OA times	1.0(0–2.0)	1.65(0–2)	1.0(0.0–3.0)	0.363
Total CA times	0(0–2.0)	0.5(0–0.25)	1.0(0–2.0)	0.048
Percentage of snoring, %	5.3(1.2–5.9)	6.6(0.1–5.7)	4.6(0–6.6)	0.447
Average SaO_2_, %	94.1(93–96)	93.7(93–95)	95.5(95.8–97)	**0.001**
Minimum SaO_2_, %	85.4(83–90)	83.9(81.2–89.0)	90.3(89.8–93.3)	**0.000**
ODI,/h	14.9 (6.1–31.9)	19.9(10.9–36.6)	2.5(1.5–4.0)	**0.000**
Average snoring time, (min)	7.4(3–10.8)	8.3(5.1–12.2)	4.2(0–7.5)	**0.015**
PLMI, /h	25.2(4.3–43.5)	25.15(4.8–41.0)	25.3(21.2–44.0)	0.733

CA, Central apnea; MSA, Multiple system atrophy; REM, Rapid eye movement; OA, Obstructive apnea; ODI, Oxygen desaturation index; PLMI, Periodic leg movement during sleep index; SE, Sleep efficiency; SL, Sleep latency; SaO_2_, Oxygen saturation; TST, Total sleep time; and WASO, Wake after sleep onset.

Values are *n* (%), mean ± SD or median (interquartile range). Bold values indicate *p* < 0.05, indicating statistically significant differences between the two groups.

Table 3 presents the process of logistic regression analysis. After adjusting for age, sex, disease duration, smoking, alcohol consumption, and MSA subtype, the associations between the presence of SRBD in MSA patients and the UMSARS score, NMSS score, HAMA score, HAMD score, MMSE score, PSQI score, and FSS score remained statistically insignificant (*p* > 0.05). It can be concluded that age, sex, disease duration, smoking, alcohol consumption, and MSA sub-type did not significantly affect the relationship between the independent and dependent variables.

**Table 3:** Risk factors of SRBD in patients with MSA.

	OR (95%CI)		
	Model 1	Model 2	Model 3
UMSARS	1.01 (0.97–1.06)^*^	1.02 (0.97–1.07)^*^	1.02 (0.97–1.08)^*^
NMSS	1.00 (0.98–1.02)^*^	1.00 (0.97–1.02)^*^	1.00 (0.97–1.03)^*^
HAMA	1.02 (0.95–1.10)^*^	1.03 (0.95–1.11)^*^	1.04 (0.95–1.13)^*^
HAMD	1.05 (0.97–1.13)^*^	1.05 (0.95–1.14)^*^	1.05 (0.96–1.15)^*^
MMSE	1.09 (0.95–1.26)^*^	1.10 (0.95–1.28)^*^	1.10 (0.93–1.29)^*^
PSQI	0.94 (0.82–1.08)^*^	0.94 (0.82–1.07)^*^	0.96 (0.83–1.12)^*^
FSS	0.94 (0.92–1.01)^*^	0.97 (0.92–1.02)^*^	0.96 (0.91–1.01)^*^

CI, confidence interval; FSS, Fatigue Severity Scale; HAMD, Hamilton Depression Scale; HAMA, Hamilton Anxiety Scale; MMSE, Mini-mental State Examination; NMSS, Non-Motor Symptoms Scale.

Model 1, Crude model; Model 2, Adjusted for age, sex; Model 3, Adjusted for age, sex, disease duration, smoking, alcohol consumption, and MSA sub-type. ^*^p > 0.05.

### Meta-analysis results

**Figure 1:**
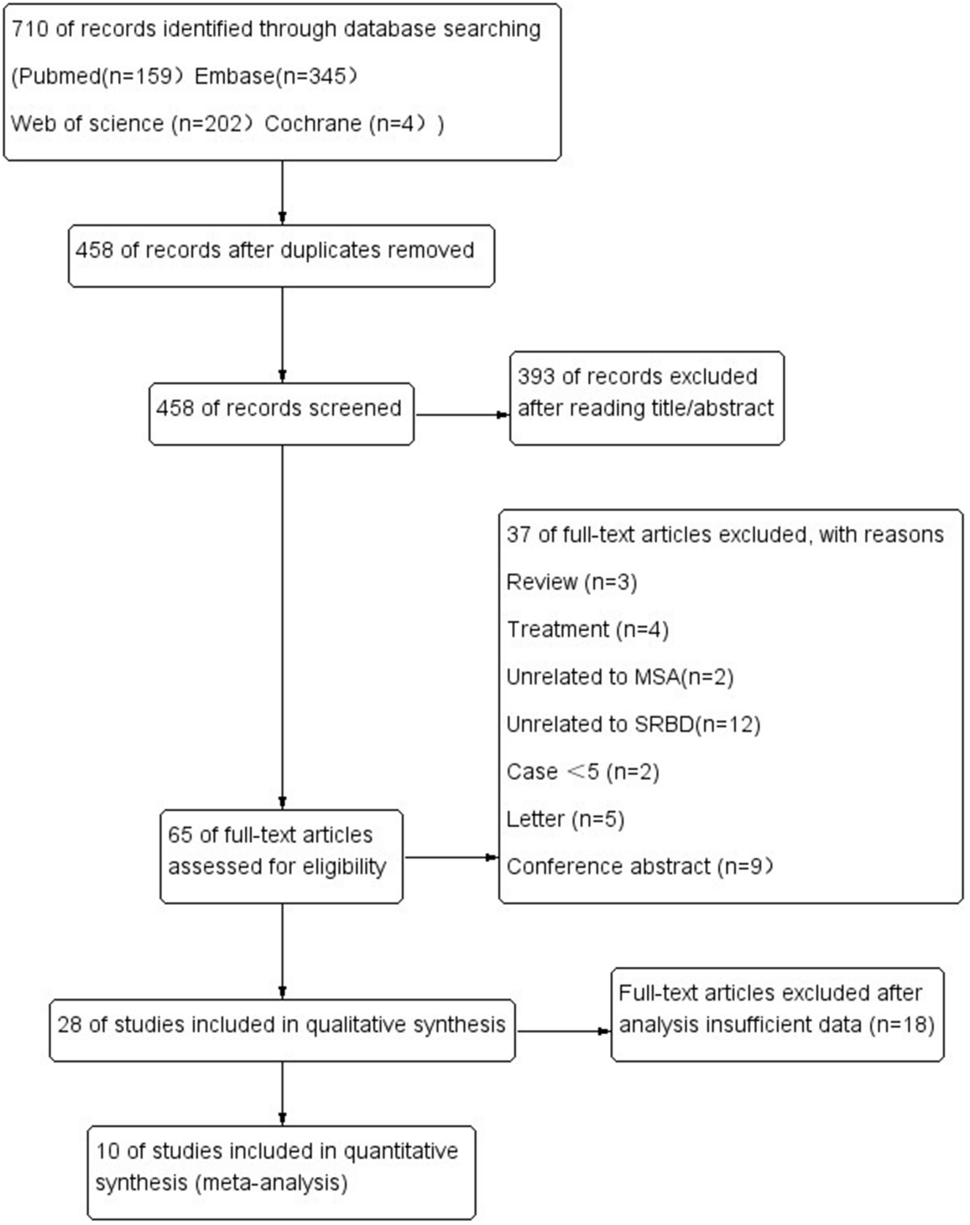
Flow diagram of systematic literature searching.

The literature search yielded 701 potentially relevant articles (Figure 1). After eliminating duplicates, 458 records were reviewed and 393 were excluded during the title and abstract screening phase. The remaining 65 full-text articles were assessed for eligibility, and 37 were excluded because they were reviews (*n* = 3), studies about treatment (*n* = 4), studies unrelated MSA (*n* = 2) or SRBD (*n* = 12), case series <5 (*n* = 2), letter (*n* = 5), conference abstract (*n* = 9), and insufficient date (*n* = 18).

In the end, 10 articles were ultimately included in our review, involving a total of 295 MSA patients. Five original researches were performed in Asia, five in Europe.

### Prevalence of SRBD

**Figure 2:**
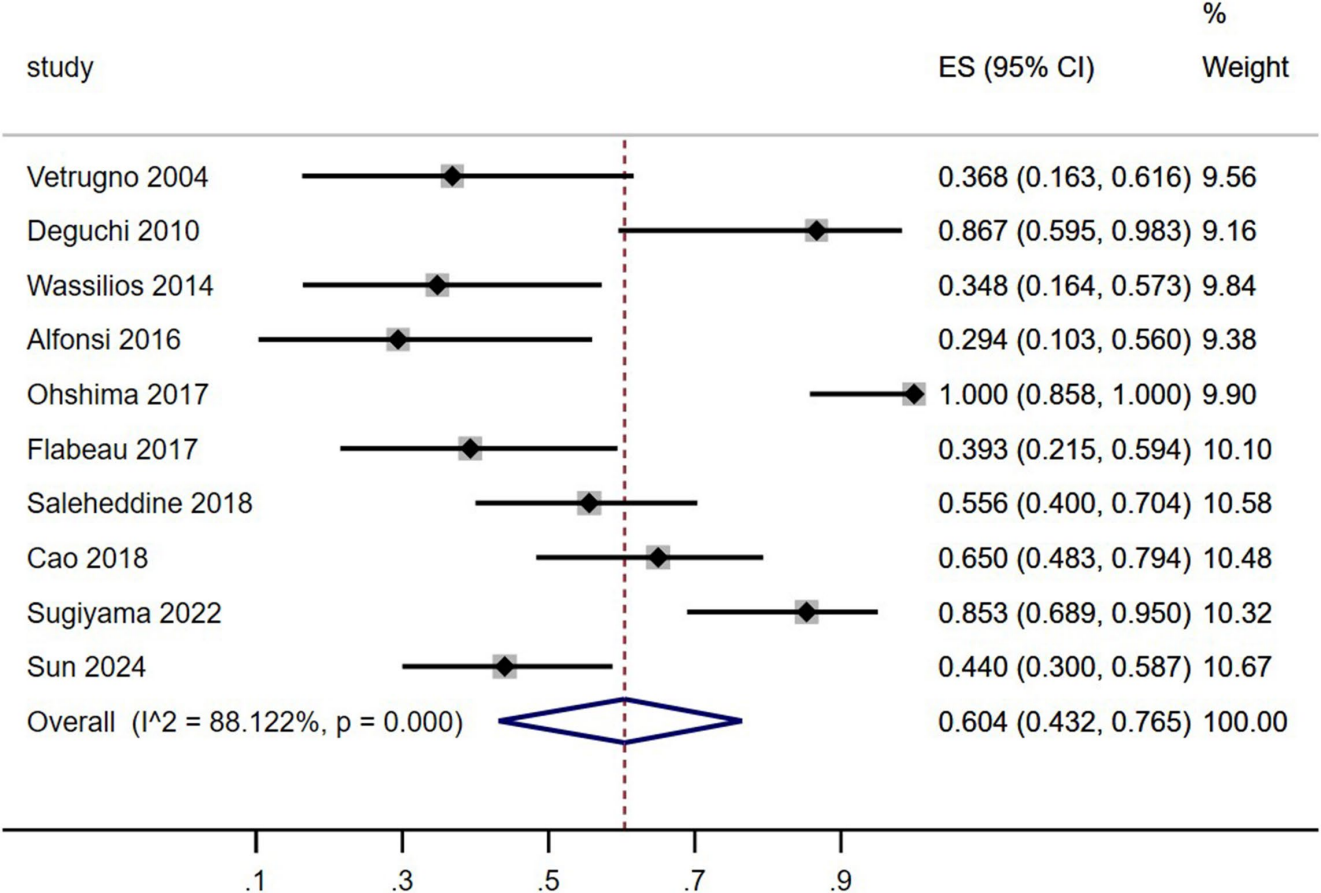
Forest plot on the pooled prevalence of sleep-related breathing disorder in multiple system atrophy.

As shown in Table 4 and Figure 2, including the results of the present study, the summary prevalence of SRBD in MSA was 60.5% (95% CI, 43.2–76.5%) in a pooled sample of 295 subjects. This study showed high heterogeneity (*I*^2^ = 88.1%). Sensitivity analysis showed unchanged results (Supplementary Figure 1.4). There was no evidence of publication bias as Begg’s and Egger’s test was not significant (Supplementary Figures 1.1.A,B).

**Table 4:** Characteristics of studies included in the meta-analyses.

References	First author	Year	Country	Sample size	Diagnostic SRBD method	Proportion of SRBD (%)	AHRQ/NOS score
(14)	Vetrugno	2004	Italy	19	PSG	37	7
(13)	Deguchi	2010	Japan	15	PSG	86.7	6
(5)	Wassilios	2014	France	23	PSG	34.8	6
(12)	Alfonsi	2016	Italy	17	PSG	29.4	7
(10)	Ohshima	2017	Japan	24	PSG	100	6
(11)	Flabeau	2017	France	28	PSG	39.3	7
(8)	Saleheddine	2018	France	45	PSG	56	7^*^
(9)	Cao	2018	China	40	PSG	65	7^*^
(7)	Sugiyama	2022	Japan	34	PSG	85	6^*^
(6)	Sun	2024	China	50	PSG	44	7
				295	PSG	60.4	

AHRQ, The Agency of Healthcare Research and Quality guideline; PSG, Polysomnography; SRBD, Sleep-related breathing disorder; MSA, Multiple system atrophy; NOS, Newcastle-Ottawa Scale.

Details of the Literature Quality Assessment scoring are shown in Supplementary Tables 1, 2. ^*^Evaluating cross-sectional studies quality using NOS.

**Figure 3:**
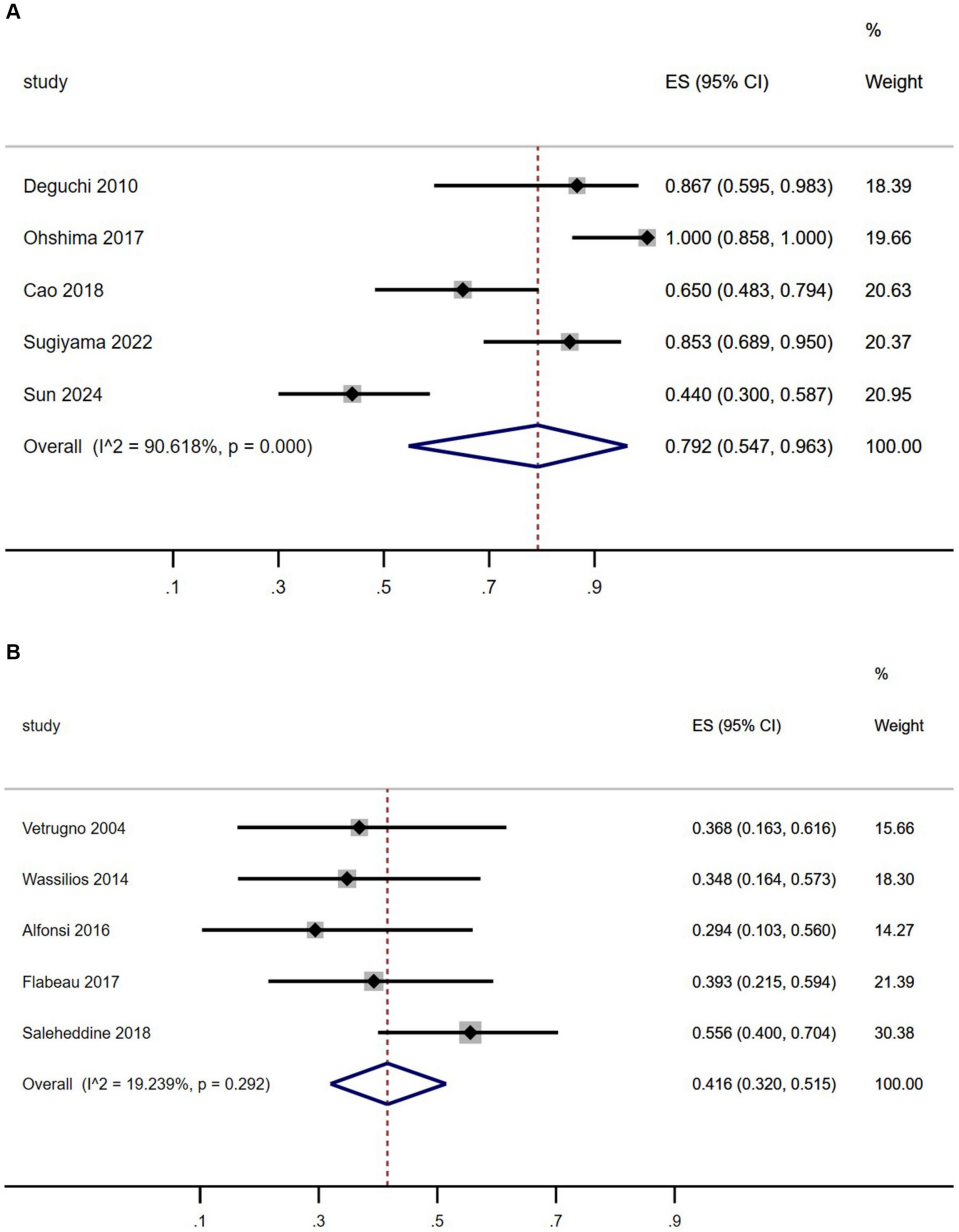
Forest plot of sleep-related breathing disorder prevalence among multiple system atrophy patients in Asian **(A)** and European **(B)** populations.

As shown in Figure 3, the summary prevalence of SRBD of MSA in Asia was 79.2% (Figure 3A, 95% CI, 54.7–96.3%), which was higher than that in Europe (Figure 3B, 41.6, 95% CI, 32–51.5%). This study revealed significant heterogeneity in the prevalence of SRBD in patients with MSA in Asia (*I*^2^ = 90.6%), while showing homogeneity in Europe (*I*^2^ = 19.2%). Sensitivity analysis confirmed consistent findings (Supplementary Figure 1.5). There was no evidence of publication bias as Begg’s and Egger’s test was not significant (Supplementary Figures 1.2.A,B, 1.3.A,B).

## Discussion

The study results revealed that 78.8% of MSA patients were diagnosed with SRBD (AHI > 5), consistent with findings from previous studies (ranging from 24.9 to 100%) (5–14). Gender prevalence did not show a significant difference, aligning with previous research (23). In terms of SRBD severity, 36.5% were classified as mild, 28.8% as moderate, and 34.6% as severe, with a slightly lower proportion of moderate cases compared to previous studies (23). A meta-analysis of previous studies indicated an overall SRBD prevalence of 60.4% in MSA patients (Table 4; Figure 2), differing from the present study’s 78.8%. Further analysis by region showed a prevalence of 79.2% in Asian MSA patients, matching the current study’s findings. In contrast, European MSA patients had a significantly lower prevalence of 41.6% (Figure 2). However, previous studies have shown that sensitivity analysis revealed a comparable pooled prevalence of SRBD (AHI > 5) in Western and Asian populations (24). Therefore, we hypothesized that the variation in SRBD prevalence between MSA patients in Asia and Europe may be attributed to differing impacts of the disease on the respiratory system in different ethnic groups. Further multicenter studies encompassing diverse regions and ethnicities are needed to validate these findings.

In this study, we compared the subjective rating scale scores of motor and non-motor symptoms in MSA patients with and without SRBD. The results indicated no significant differences between the two groups in terms of age, sex, disease onset, disease duration, severity of motor symptoms (UMSARS I, II, IV), severity of non-motor symptoms (NMSS), anxiety and depression levels (HAMA, HAMD), daytime sleepiness (ESS), fatigue (FSS), cognitive functioning (MMSE), and sleep quality (PSQI). These findings suggest that SRBD is not linked to the severity of motor and non-motor symptoms, including cognition, daytime sleepiness, fatigue, and sleep quality in MSA patients. However, previous studies have reported conflicting results, showing that MSA patients with SRBD had higher UMSARS scores and more severe depressive symptoms compared to those without SRBD (9). This discrepancy could be attributed to the limited number of patients (*n* = 40) in the previous study (9), highlighting the need for larger multicenter studies to confirm our conclusions.

In patients with MSA, SRBD encompass obstructive sleep apnea (OSA) and central sleep apnea (CSA). This study revealed that MSA patients with SRBD had a higher frequency and index of obstructive apnea during sleep compared to central apnea. Conversely, MSA patients without SRBD showed similar frequencies and indices of obstructive and central apnea during sleep, aligning with findings from a previous study (9). Furthermore, MSA patients with SRBD exhibited a higher index of obstructive apnea during sleep than those without SRBD, while the index of central apnea did not significantly differ between MSA patients with or without SRBD, consistent with previous research (9). Previous research has linked OSA to abnormal upper airway anatomy, compromised stability of ventilatory control, and dysfunction in upper airway muscles and their neuromodulation (25). These underlying mechanisms result in hypoventilation during sleep in affected individuals. Our study revealed that MSA patients with SRBD exhibited a higher hypopnea index and a greater percentage of snoring during sleep compared to MSA patients without SRBD. Central apnea in MSA may be linked to the loss of neuronal cells in specific respiratory rhythm-controlling areas of the brain (26). Therefore, SRBD in MSA patients is likely due to obstructive apnea from abnormal upper airway caused by the disease itself, rather than central apnea from central nervous system atrophy. Our study found no significant differences in total sleep time, sleep efficiency, sleep structure, or subjective sleep quality between MSA patients with and without SRBD, consistent with previous research (9). However, patients with MSA and SRBD exhibited lower mean oxygen saturation and higher oxygen desaturation index during sleep compared to those without SRBD. Previous studies have also indicated a higher prevalence of excessive daytime sleepiness (EDS) in MSA patients with SRBD (9). This suggests that SRBD may contribute to the development of EDS in these patients. By synthesizing our findings with existing literature, we propose that SRBD does not impact sleep quality or architecture in MSA patients, and that the increased EDS in MSA patients with SRBD may be attributed to the hypoxic nature of SRBD during sleep.

The study’s strengths included a larger sample size, low dropout rate, and comprehensive clinical and PSG variables. However, several limitations should be acknowledged. Firstly, the average age of the participants was 60 years, suggesting a potential risk of selection bias. Secondly, neurodegenerative disease diagnosis relied on clinical assessment, potentially leading to diagnostic accuracy issues. Thirdly, all patients had to meet the diagnostic criteria for probable MSA, introducing a selection bias towards more severe cases. Fourthly, non-motor symptoms were mainly assessed through questionnaires, potentially affecting diagnostic precision. Fifthly, the study was conducted at a single center, highlighting the need for multicenter studies involving diverse populations to determine SRBD prevalence in MSA patients. Sixthly, both our study and existing literature on SRBD effects in MSA patients have primarily originated from Asia, with a lack of studies from Europe and the United States. This geographical bias raises questions about potential ethnic differences in SRBD effects on MSA. Additionally, the criteria used to diagnose probable MSA are those published in 2008, not the most recent criteria published by the Movement Disorder Society in 2022.

## Conclusion

The study found a prevalence of 78.8% of SRBD in MSA patients, with a notably higher prevalence in Asia compared to Europe. The majority of SRBD cases in MSA were attributed to obstructive apnea. Furthermore, the presence of SRBD did not show a significant impact on the motor and non-motor symptoms of MSA patients.

## Data Availability

The original contributions presented in the study are included in the article/Supplementary material, further inquiries can be directed to the corresponding author.
