# Supplementary figures and images for: Prevalence and impact of sleep-related breathing disorder in multiple system atrophy patients: a cross-sectional study and meta-analysis
Source: Front Neurol. 2024 Aug 20;15:1440932. doi: 10.3389/fneur.2024.1440932 (PMC11368784; doi:10.3389/fneur.2024.1440932)

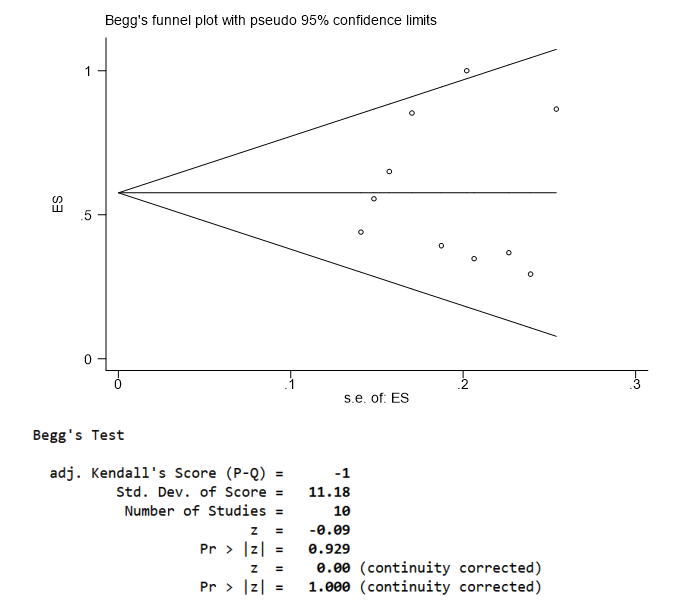

Supplement: Supplementary file 1 [file Image_1.PNG]

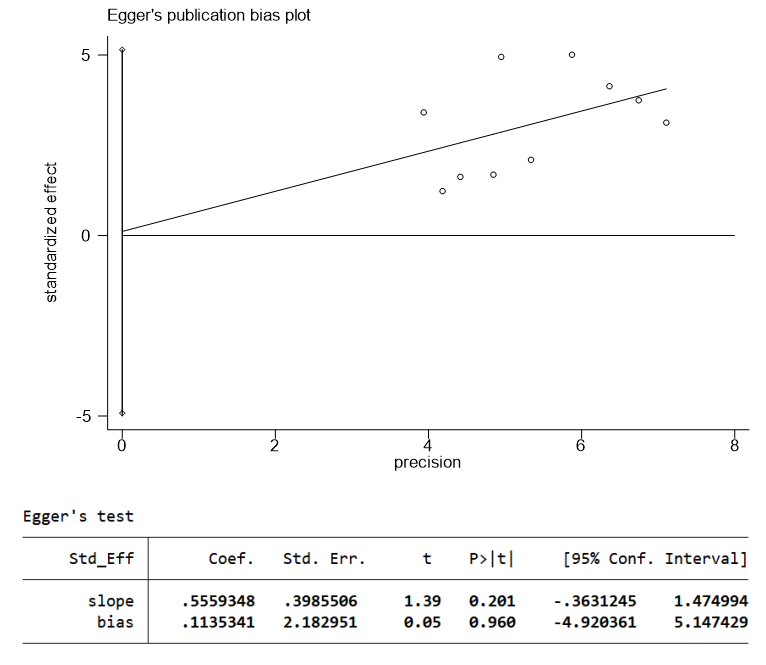

Supplement: Supplementary file 2 [file Image_2.PNG]

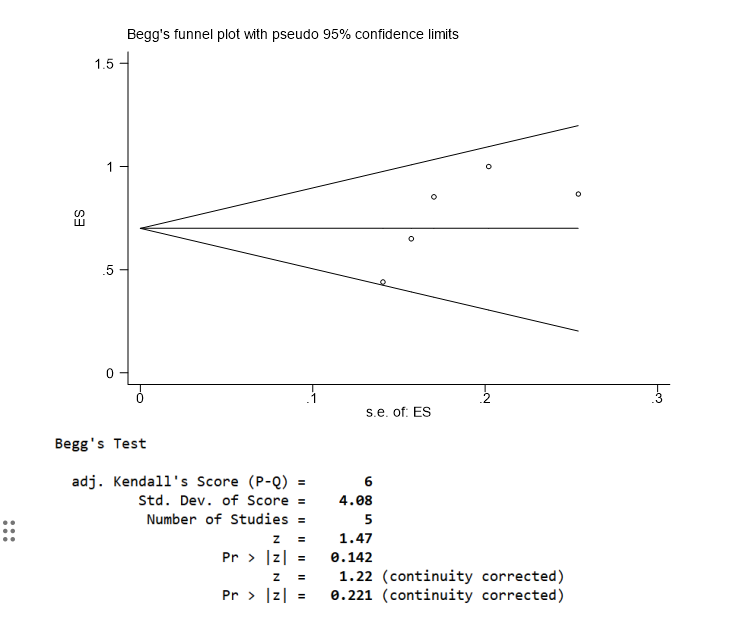

Supplement: Supplementary file 3 [file Image_3.PNG]

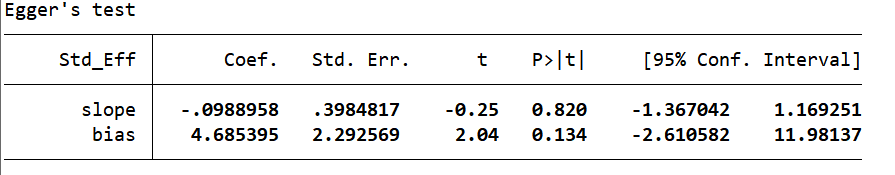

Supplement: Supplementary file 4 [file Image_4.PNG]

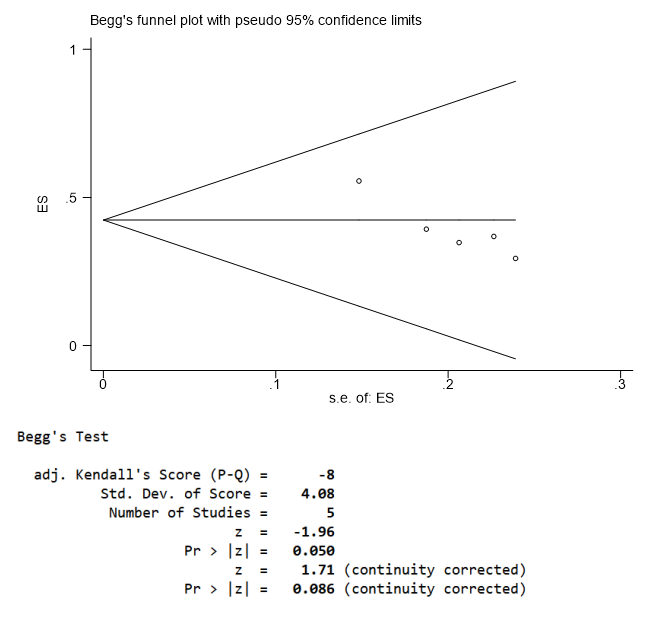

Supplement: Supplementary file 5 [file Image_5.PNG]

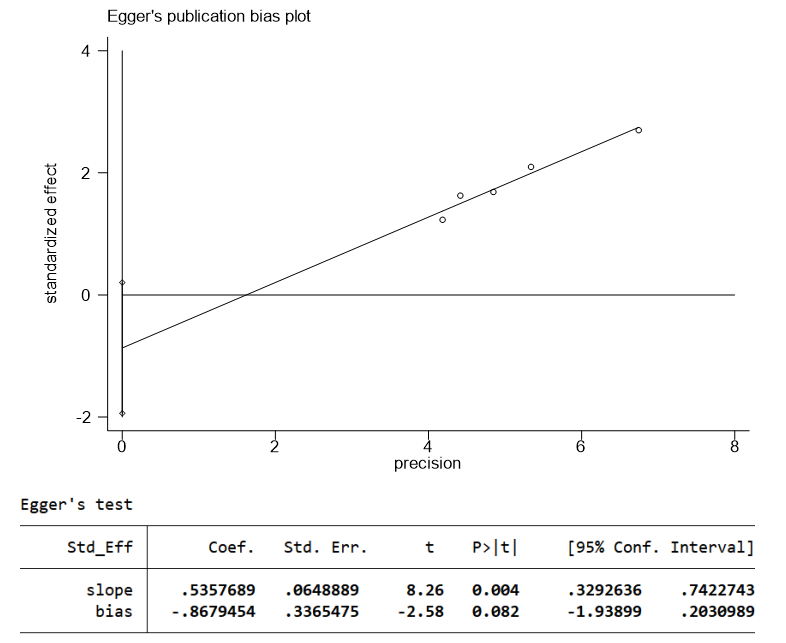

Supplement: Supplementary file 6 [file Image_6.PNG]

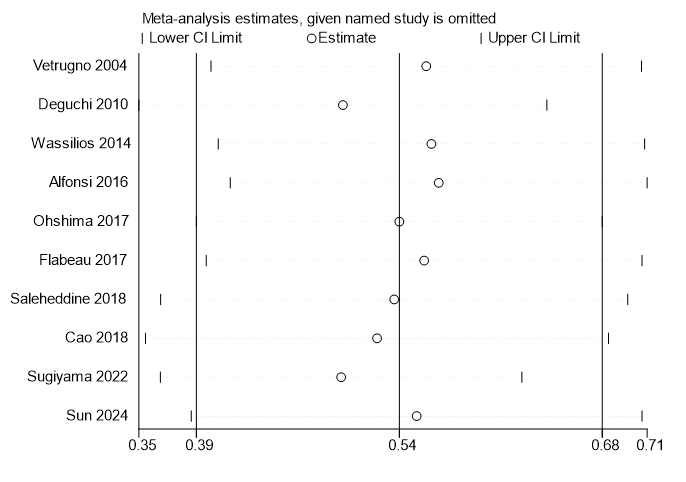

Supplement: Supplementary file 7 [file Image_7.PNG]

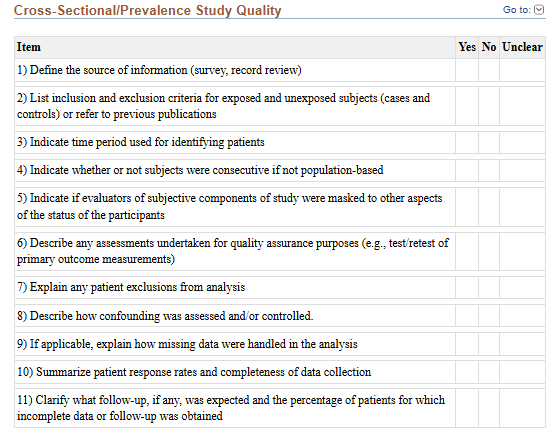

Supplement: Supplementary file 8 [file Image_8.PNG]

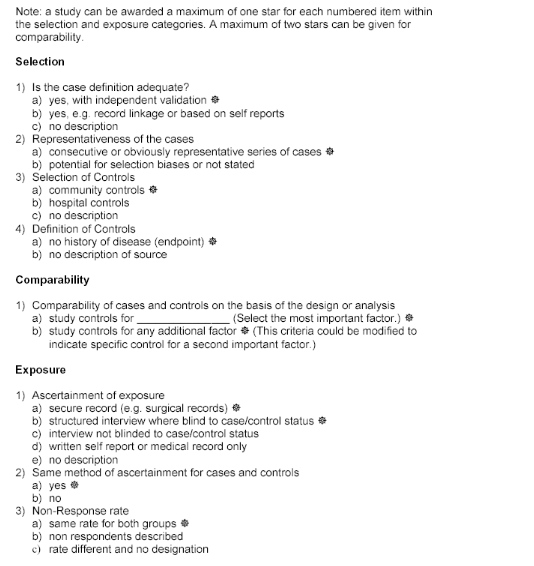

Supplement: Supplementary file 9 [file Image_9.PNG]
