# Supplementary material for: Prevalence and impact of sleep-related breathing disorder in multiple system atrophy patients: a cross-sectional study and meta-analysis
Source: Front Neurol. 2024 Aug 20;15:1440932. doi: 10.3389/fneur.2024.1440932 (PMC11368784; doi:10.3389/fneur.2024.1440932)
Supplement: Supplementary file 10 [file Table_1.docx]

**Table 1.Evaluating studies using NOS**

| **Reference** | **First author** | **Year** | **Selection** | | | | **Comparability** | | **Eposure** | | | **Total score** |
| --- | --- | --- | --- | --- | --- | --- | --- | --- | --- | --- | --- | --- |
| [15] | Flabeau | 2017 | 0.5 | 0.5 | 1 | 1 | 0.5 | 0.5 | 1 | 1 | 1 | 7 |
| [12] | Saleheddine | 2018 | 0.5 | 0.5 | 1 | 1 | 0.5 | 0.5 | 1 | 1 | 1 | 7 |
| [8] | Sugiyama | 2022 | 0.5 | 0.5 | 1 | 1 | 0.5 | 0.5 | 1 | 0 | 1 | 6 |

**Abbreviations: NOS**,Newcastle-Ottawa Scale
