# Supplementary material for: Prevalence and impact of sleep-related breathing disorder in multiple system atrophy patients: a cross-sectional study and meta-analysis
Source: Front Neurol. 2024 Aug 20;15:1440932. doi: 10.3389/fneur.2024.1440932 (PMC11368784; doi:10.3389/fneur.2024.1440932)
Supplement: Supplementary file 11 [file Table_2.docx]

**Table 2.Evaluating studies using AHRQ**

| **Reference** | **First author** | **Year** | **Item 1** | **Item 2** | **Item 3** | **Item 4** | **Item 5** | **Item 6** | **Item 7** | **Item 8** | **Item 9** | **Item 10** | **Item 11** | **Total score** |
| --- | --- | --- | --- | --- | --- | --- | --- | --- | --- | --- | --- | --- | --- | --- |
| [19] | Vetrugno | 2004 | 1 | 0 | 1 | 1 | 0 | 0 | 1 | 1 | 1 | 1 | 0 | 7 |
| [17] | Deguchi | 2010 | 1 | 0 | 1 | 1 | 1 | 0 | 0 | 0 | 1 | 0 | 0 | 6 |
| [6] | Wassilios | 2014 | 1 | 0 | 1 | 1 | 0 | 0 | 1 | 1 | 1 | 0 | 0 | 6 |
| [16] | Alfonsi | 2016 | 1 | 0 | 1 | 1 | 0 | 0 | 1 | 1 | 1 | 1 | 0 | 7 |
| [14] | Ohshima | 2017 | 1 | 0 | 1 | 1 | 0 | 1 | 0 | 1 | 0 | 1 | 0 | 6 |
| [13] | Cao | 2018 | 1 | 1 | 1 | 1 | 0 | 0 | 1 | 1 | 0 | 1 | 0 | 7 |
| [7] | Sun | 2024 | 1 | 1 | 1 | 1 | 0 | 0 | 1 | 1 | 1 | 0 | 0 | 7 |

**Abbreviations:AHRQ**, the Agency of Healthcare Research and Quality guideline

1 point for each item if it is a yes, 0 points if it is a “no” or not described in the study.
